# Alteration of transthyretin and thyroxine-binding globulin in major depressive disorder: multiple reaction monitoring-based proteomic analysis

**DOI:** 10.1186/s12967-021-02702-y

**Published:** 2021-01-15

**Authors:** Hye In Woo, Jisook Park, Shinn-Won Lim, Doh Kwan Kim, Soo-Youn Lee

**Affiliations:** 1 Department of Laboratory Medicine, Samsung Changwon Hospital, Sungkyunkwan University School of Medicine, Changwon, Korea; 2 Samsung Biomedical Research Institute, Samsung Medical Center, Sungkyunkwan University School of Medicine, Seoul, Korea; 3 SAIHST, Sungkyunkwan University School of Medicine, Samsung Medical Center, Seoul, Korea; 4 Department of Psychiatry, Samsung Medical Center, Sungkyunkwan University School of Medicine, #81 Irwon-ro, Gangnam-gu, Seoul, 06351 Korea; 5 Department of Laboratory Medicine and Genetics, Samsung Medical Center, Sungkyunkwan University School of Medicine, #81 Irwon-ro, Gangnam-gu, Seoul, 06351 Korea; 6 Department of Clinical Pharmacology and Therapeutics, Samsung Medical Center, Sungkyunkwan University School of Medicine, Seoul, Korea

**Keywords:** MDD, Selective serotonin reuptake inhibitors, SSRI, Mirtazapine, Proteomics, Thyroid

## Abstract

**Background:**

Major depressive disorder (MDD), common mental disorder, lacks objective diagnostic and prognosis biomarkers. The objective of this study was to perform proteomic analysis to identify proteins with changed expression levels after antidepressant treatment and investigate differences in protein expression between MDD patients and healthy individuals.

**Methods:**

A total of 111 proteins obtained from literature review were subjected to multiple reaction monitoring (MRM)-based protein quantitation. Finally, seven proteins were quantified for plasma specimens of 10 healthy controls and 78 MDD patients (those at baseline and at 6 weeks after antidepressant treatment of either selective serotonin reuptake inhibitors (SSRIs) or mirtazapine).

**Results:**

Among 78 MDD patients, 35 patients were treated with SSRIs and 43 patients were treated with mirtazapine. Nineteen (54.3%) and 16 (37.2%) patients responded to SSRIs and mirtazapine, respectively. Comparing MDD patients with healthy individuals, alteration of transthyretin was observed in MDD (P = 0.026). A few differences were observed in protein levels related to SSRIs treatment, although they were not statistically significant. Plasma thyroxine-binding globulin (TBG) was different between before and after mirtazapine treatment only in responders (P = 0.007).

**Conclusions:**

In proteomic analysis of plasma specimens from MDD patients, transthyretin and TBG levels were altered in MDD and changed after antidepressant treatment.

## Background

Major depressive disorder (MDD) is a psychiatric disorder with heterogeneous symptoms, including distinct change of mood, sadness, and psychophysiological changes sleep and appetite disturbances [1]. Globally, MDD affects approximately 5–20% of the world population [2, 3].

Many previous studies have reported that patients with MDD show various kinds of biological disturbance, including abnormal functioning of neurotransmitters [4], inflammatory changes [5], and dysregulation of the endocrine system that include a dysregulated hypothalamic–pituitary–adrenal axis [6, 7] and reproductive endocrine changes [8]. However, etiology and consequences of MDD remain unclear due to innate complexity of MDD and multifactored etiologies such as genetic and environmental factors including trauma during childhood or adulthood and stressful life events [9]. Currently there are no firm biomarkers for diagnosis of MDD [10], leading to the lack of objective diagnostic tool and misdiagnosis of MDD, especially in primary care setting [10]. Although at least one-third of patients treated with second-generation antidepressants do not achieve treatment responses [11, 12], there is no relevant biomarker to predict antidepressant treatment response either.

To identify biomarkers of MDD, many studies have been conducted in diverse research fields, including genomics, transcriptomics, proteomics, and metabolomics [13–16]. Especially, proteomic approach is considered a promising tool because proteins account environmental influence and closely reflect the pathophysiologic process of psychiatric conditions [17]. To discover diagnostic biomarkers for MDD using proteomic approach, several clinical studies have been performed using brain tissue [18, 19], cerebrospinal fluid (CSF) [20], urine [21], and serum/plasma [22–24]. These studies have reported protein expression alterations in lipid metabolism and immunoregulation [23, 25], inflammatory response [26], oxidative stress response, growth factor pathway [24], neuroprotection, and neuronal development [20] in MDD compared to healthy individuals.

A few proteomic studies related to prediction of treatment response and changes by antidepressant treatment have been reported, including increase of pigment epithelium-derived factor level after electroconvulsive therapy (ECT) [27], decrease of proteasome subunit α type-2 in antidepressant responders [28], and association of apolipoprotein A-IV with antidepressant response [29]. However, these studies are focused on changes of proteins according to mixed antidepressants [28, 29] and ECT [27], or on proteins involved in immune, endocrine, and metabolic processes [29]. More proteomic studies using precise analytic method to identify predictive protein markers, especially peripheral blood markers, are required because they are non-invasive and practical for clinical use [10].

Therefore, the objective of this study was to perform multiple reaction monitoring (MRM)-based proteomic analysis to identify proteins whose expression levels were changed after antidepressant treatment, that might be able to predict treatment response, and assess differences in protein expression between MDD patients and healthy individuals. We screened 111 proteins using MRM method and performed quantitative proteomic analysis usingstable isotope labeled peptide in plasma from MDD patients. To the best of our knowledge, this is the first report describing changes in protein levels after antidepressant treatment in MDD patients using MRM-based proteomic analysis.

## Methods

### Patients

MDD patients fulfilling the Diagnostic and Statistical Manual of Mental Disorders, Fourth Edition, criteria for major depressive episode who were treated with selective serotonin reuptake inhibitors (SSRIs: escitalopram, fluoxetine, paroxetine, sertraline), or non-SSRI (mirtazapine) were recruited. Diagnosis was confirmed by board certified psychiatrists based on Samsung Psychiatric Evaluation Schedule, case review notes, and SCID (structured clinical interview for DSM-IV) to diagnose depression. A minimum baseline score of 15 for 17-item Hamilton Rating Scale for Depression (HAM-D) was required [30]. Study participants were excluded if they had pregnancy, significant medical conditions, abnormal laboratory baseline values, unstable psychiatric features (e.g., suicidal), history of alcohol or drug dependence, seizures, head trauma with loss of consciousness, neurological illness, or concomitant Axis I psychiatric disorder.

Patients received monotherapy for 6 weeks with one of four commonly used SSRI or mirtazapine antidepressants by clinician’s choice. In this study, choice of drug was driven by the preference of the physician, with consideration of anticipated side effects in at-risk individuals. Dose titration was completed within two weeks. Trough plasma samples were drawn at the end of week 6 for plasma drug concentrations. Lorazepam 0.5–1 mg was allowed at bedtime for insomnia. Patients were seen by a psychiatrist, who monitored their adverse events and severity of depression. The HAM-D was administered by a single trained rater every two weeks [30]. The rater and personnel of protein quantitation were blinded to the hypotheses, drug assignment, and HAM-D data. Therapeutic response was defined as 50% or more reduction of HAM-D score by 6 weeks after initiation of antidepressant treatment.

Clinical data of depressant patients including age, gender, HAM-D score, antidepressant, family history, onset age, number of episode, and duration of current episode were collected for each individual. Healthy individuals without known past medical or psychiatric history or family history of MDD were included as controls. This study was approved by Samsung Medical Center Institutional Review Board. Informed consent was obtained from all participants.

**Fig. 1:**
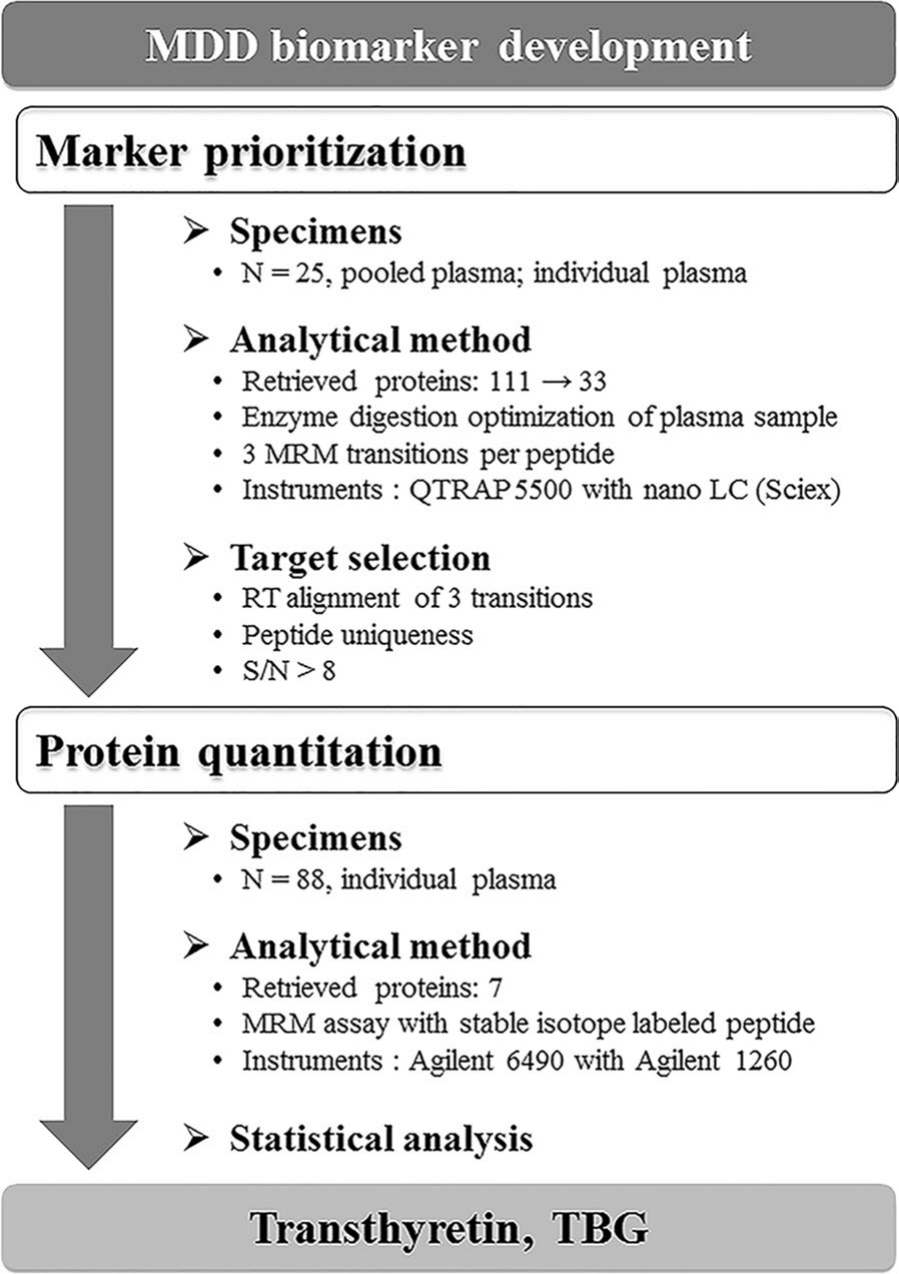
Overall scheme of proteomic analysis. Plasma specimens were collected before and at 6 weeks after antidepressant treatment in each patient group and healthy individuals. *MRM* multiple reaction monitoring, *MS* mass spectrometry, *N* number of patients, *RT* retention time, *S/N* signal-to-noise ratio, *TBG* thyroxine-binding globulin

Peripheral blood specimens were obtained from patients with MDD at baseline and at 6 weeks after initiation of antidepressant treatment. Patients were categorized into four groups according to antidepressant type used and treatment responsiveness: SSRIs responder, SSRIs nonresponder, mirtazapine responder, and mirtazapine nonresponder. For marker prioritization, we prepared pooled plasma by pooling equal amounts of plasma specimen from five individuals for each group. To investigate whether proteins detected in pooled plasma were detectable in each plasma, we used these 45 specimens individually. For protein quantitation, we used additional 166 plasma specimens at baseline and at 6 weeks after initiation of either SSRIs or mirtazapine treatment from 78 MDD patients and 10 healthy controls (Fig. 1).

### Materials

NuPAGE gels (4 ~ 12%) were acquired from Invitrogen and PIERCE (Rockford, IL, USA). Sequencing-grade modified trypsin was purchased from Promega (Madison, WI, USA). Acetonitrile (ACN; MS grade), water (MS grade), and formic acid (FA; ACS regent grade) were acquired from Aldrich (Milwaukee, WI, USA). Seven isotope-labeled peptides were synthesized as internal standards for MRM assay (AnyGen Co., Gwangju, Korea): TEDTIFL*R, VANYVDWI*NDR, ITLPDFTGDL*R, ELLESYI*DGR, VSLATV*DK, NALALFVLP*K, and AADDTWEP*FASGK for alpha-1-acid glycoprotein 1 (AGP1), hepatocyte growth factor activator (HGFA), lipopolysaccharide-binding protein (LBP), prothrombin, selenoprotein P (SeP), thyroxine-binding globulin (TBG), and transthyretin, respectively. * represents amino acid labeled with ^13^C^15^N heavy isotope.

### Marker surrogate selection

We searched proteins that were either related to depressive disorders or located on antidepressant action sites or metabolic pathways in previous individual studies and meta-analysis. In addition to protein markers, genetic markers, DNA, and mRNA were also selected. We manually reviewed them and obtained proteins encoded by genes as well as proteins described in previous literatures. Among selected proteins, we gave priorities to proteins present in serum or plasma based on public databases including Healthy Human Individuals Integrated Plasma Proteome Database and Sys-Body Fluid database as they were studied much more in literatures. Finally, a total of 111 proteins were selected for MRM-based marker prioritization (Additional file 1: Table S1).

### Sample preparation

For marker prioritization, 50 μL of plasma was resolved on 4 ~ 12% NuPAGE gel which was cut into 10 bands and subjected to in-gel tryptic digestion prior to MRM analysis. In-gel digestion was achieved at a 50:1 ratio for 16 h at 37 °C. Tryptic digests were recovered by extraction with 50% ACN/0.1% FA and purified using an OMIX C-18 tip (Agilent, Technologies, Santa Clara, CA, USA).

For protein quantitation, 1 μL of plasma was mixed with 40 μL of 10 mM of dithiothreitol and incubated at 60 °C for 45 min for protein reduction. Next, 5 mM iodoacetamide was added. Samples were incubated at room temperature for 30 min in the dark to induce alkylation followed by digestion with trypsin (1:50) overnight at 37 °C, after which digested samples were dried using a centrifugal evaporator. Each tryptic digest sample was spiked with stable isotope labeled peptide. Samples were dried and reconstituted with 20 μL of 0.1% FA in water.

### MRM-based marker prioritization

We generated MRM transitions using MRMPilot™ v2.0 (AB Sciex, Framingham, MA, USA) against 111 proteins selected by literature review. We then monitored these MRM methods using pooled plasma. We performed MRM analysis using a QTRAP 5500 hybrid triple quadrupole/linear ion trap mass spectrometer (AB Sciex) equipped with a nano-electrospray ion source. MRM mode setting was as follows: curtain gas and spray gas at 20 and 25 psi, respectively; collision gas set on high level; and declustering potential set at 100 V. Among 111 proteins, 33 proteins showed signal-to-noise ratio (S/N) above 8 in pooled plasma specimen. For these 33 proteins, we performed MRM analysis using individual plasma specimens to select marker candidates for protein quantitation using stable isotope labeled peptide. The MRM method was identical to that was used for protein measurement of pooled plasma.

### MRM-based protein quantitation

Through marker prioritization, seven proteins were detectable (S/N > 8) in individual plasma. For these seven proteins, we performed protein quantitation using stable isotope labeled peptide. Each tryptic digest sample was spiked with stable isotope labeled peptide. Samples were dried and reconstituted with 20 μL of 0.1% FA in water. MRM analysis was performed using Agilent 6490 Triple Quadrupole mass spectrometer equipped with Agilent 1260 Infinity LC system (Agilent Technologies Inc., Table 1). Tryptic peptides were loaded onto a reversed phase analytical column (150 mm × 0.2 mm i.d., Agilent ZORBAX Eclipse Plus, 1.6 μm particle size) that was maintained at a column temperature of 40 °C. Sample separations were achieved using mobile phase A consisting of 0.05% FA and 0.2% methanol in water and mobile phase B consisting of 0.05% FA and 0.2% methanol in ACN. The gradient method was composed of multiple linear gradients as follows (time, % B, flow rate): 3 min, 2% B, 0.25 mL/min; 43 min, 30% B, 0.25 mL/min; 47 min, 90% B, 0.27 mL/min; 53.1 min, 1% B, 0.25 mL/min. Separated peptides were ionized using positive electrospray ionization: 3500 V capillary voltage, 150 V (high pressure RF) and 60 V nozzle voltage (low pressure RF), a sheath gas flow of 11 L/min at a temperature of 200 °C, a drying gas flow rate of 16 L/min at a temperature of 150 °C, and 30 psi nebulizer gas flow.

**Table 1:** Proteins for quantitative analysis using stable isotope labeled peptides

Protein full name	Short name	Peptide sequence	Targetion	Intact		IS		CE
				Q1	Q3	Q1	Q3	
Alpha-1-acid glycoprotein 1	AGP1	TEDTIFL*R	+ 2y6	497.8	764.4	501.3	771.4	16
Hepatocyte growth factor activator	HGFA	VANYVDWI*NDR	+ 2y7	682.8	917.4	686.3	924.5	22
Lipopolysaccharide-binding protein	LBP	ITLPDFTGDL*R	+ 2y8	624.3	920.4	627.8	927.5	20
Prothrombin	Prothrombin	ELLESYI*DGR	+ 2y6	597.8	710.3	601.3	717.4	19
Selenoprotein P	SeP	VSLATV*DK	+ 2y6	416.7	646.4	419.7	652.4	10
Thyroxine-binding globulin	TBG	NALALFVLP*K	+ 2y7	543.3	787.5	546.3	793.5	13
Transthyretin	Transthyretin	AADDTWEP*FASGK	+ 2y6	697.8	606.3	700.8	612.3	26

*CE* collision energy, *IS* internal standard

### Statistical analysis

To identify proteins differentially expressed between MDD patients and healthy individuals and between responders and nonresponders at baseline, we performed Fisher’s exact test for categorical variables and t-test or Mann–Whitney U test for continuous variables. Comparisons between patients at baseline and at 6 weeks after initiation of antidepressants in total and subset of patients were performed using Wilcoxon signed rank test or paired t-test. Each comparison was independently performed for SSRIs and mirtazapine groups. Proteins and clinical variables with univariate p-values less than 0.200 were included in multivariate analysis using partial Spearman correlation analysis. P-value of less than 0.050 was regarded as statistically significant. SAS version 9.3 (SAS Institute, Cary, NC, USA) was used for all statistical analyses.

## Results

### Patient characteristics and protein quantitation

Clinical and demographic characteristics of MDD patients at baseline and healthy individuals are summarized in Table 2. Age, gender, HAM-D score, onset age, number of episode, duration of current episode, and antidepressant used in study population were investigated. Overall, SSRIs- or mirtazapine-treated patients and healthy individuals and MDD patients showed no statistically significant differences. Responders and nonresponders did not differ in any variables at baseline. Among 111 proteins selected by literature review, 33 proteins showed S/N ratio above 8 in pooled plasma specimen. Among these 33 proteins, seven proteins (AGP1, HGFA, LBP, prothrombin, SeP, TBG, and transthyretin) showed S/N ratio above 8 in individual plasma specimens. For these seven proteins, we performed quantitative analysis using stable isotope labeled peptide in 166 plasma specimens from 78 MDD patients and 10 healthy individuals and compared protein expressions for the following groups: patients vs. controls, responders vs. nonresponders, and at baseline vs. at 6 weeks after treatment.

**Table 2:** Clinical characteristics of major depressive patients (n = 78) at baseline and healthy controls (n = 10)

Characteristics	Control	SSRIs		Mirtazapine		P
		R	NR	R	NR	
N	10	19	16	22	21	
Age, mean ± SD, year	67.4 ± 6.7	65.9 ± 12.3	62.4 ± 10.2	66.4 ± 8.8	66.8 ± 9.4	0.884
Gender, M:F, n	3:7	6:13	4:12	6:16	6:15	0.996
HAM-D score, mean ± SD		19.0 ± 3.11	20.6 ± 2.76	18.2 ± 2.64	20.2 ± 3.79	0.320
Onset age, mean ± SD, year		56.4 ± 14.2	57.6 ± 9.66	56.5 ± 15.6	55.1 ± 14.5	0.573
No. of episode, mean ± SD		2.58 ± 1.92	1.75 ± 0.86	2.64 ± 2.06	3.33 ± 2.83	0.376
Duration of current episode, mean ± SD, m		4.21 ± 3.60	9.56 ± 7.89	3.86 ± 3.12	7.19 ± 6.59	0.105
Antidepressant used, n (%)						0.956
Escitalopram		2 (10.5)	1 (6.3)			
Fluoxetine		8 (42.1)	9 (56.3)			
Paroxetine		5 (26.3)	3 (18.8)			
Sertraline		4 (21.1)	3 (18.8)			
Mirtazapine				22	21	

*HAM-D* Hamilton Depression Rating Scale, *NR* nonresponders; *R* responder; *SD* standard deviation, *SSRIs* selective serotonin reuptake inhibitors

### Differentially expressed proteins between MDD patients and controls

**Fig. 2:**
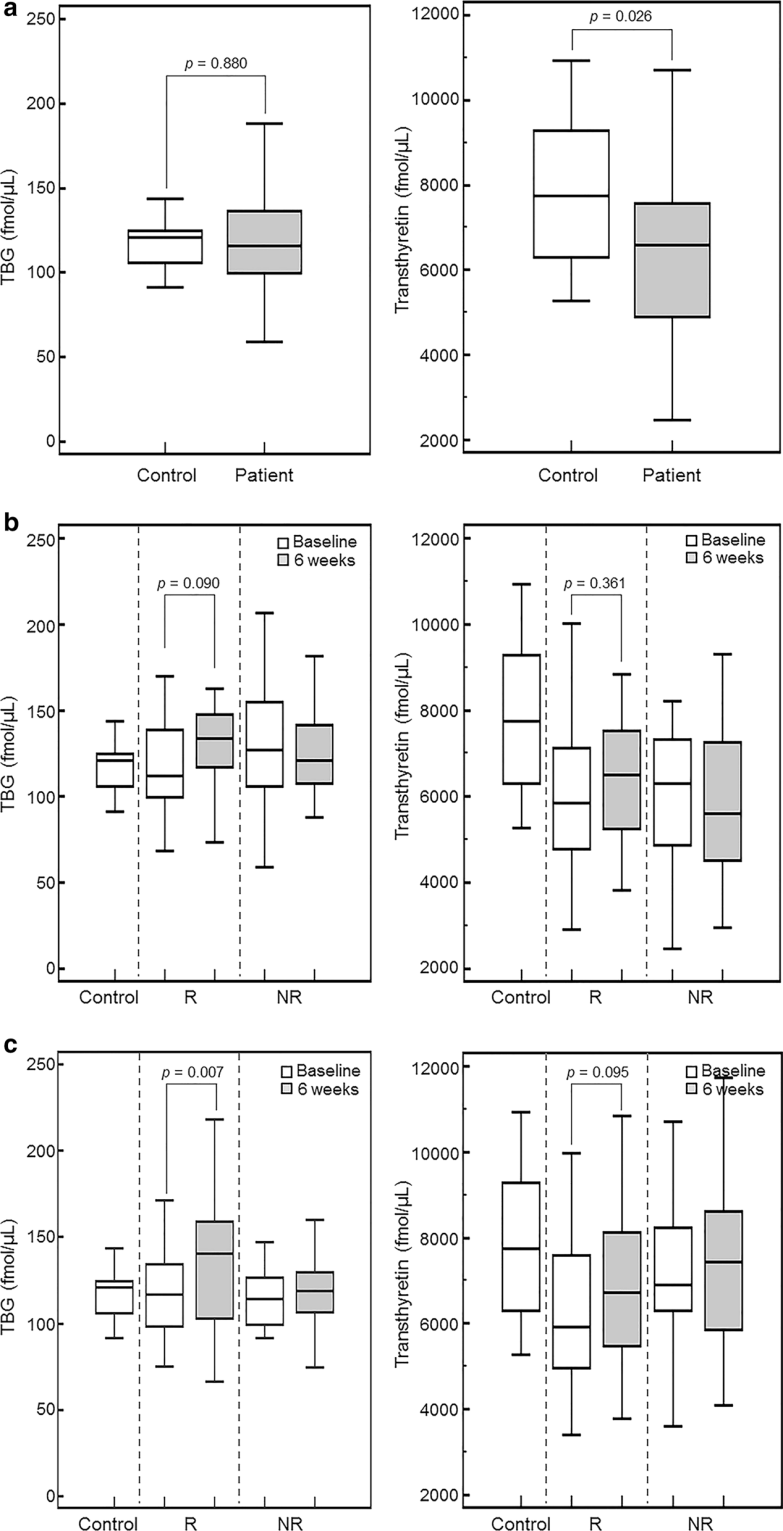
Plasma levels of thyroxine-binding globulin (TBG) and transthyretin in healthy controls and major depressive disorder (MDD) patients treated with either selective serotonin reuptake inhibitors (SSRIs) or mirtazapine. **a** Comparison between healthy individuals and patients with MDD. Transthyretin was higher in controls than that in MDD patients. **b** Plasma protein levels in MDD patients treated with SSRIs. TBG levels were increased in responders after SSRIs treatment. However, the difference did not reach statistical significances. **c** Plasma protein levels in MDD patients treated with mirtazapine. TBG level was increased after mirtazapine treatment. Transthyretin levels tended to be lower in responders. However, this difference did not reach the statistical significance. NR, nonresponder; R, responder

After comparison between patients at baseline and controls, transthyretin showed lower concentration in MDD patients than that in controls (mean, 6338 vs. 7718 fmol/μL, uni-P = 0.026, Table 3 and Fig. 2a). This statistical significance was maintained after adjusting for age and gender (multi-P = 0.038). Although AGP1 concentration was also lower in MDD patients than that in controls, such difference did not reach statistical significance (7874 vs. 9448 fmol/μL, uni-P = 0.116).

**Table 3:** Plasma protein concentrations (fmol/μL) between major depressive patients at baseline and healthy controls

Protein	Mean ± SD		Uni-P	Multi-P^a^
	Control	MDD		
N	10	78		
AGP1	9448 ± 3091	7874 ± 3063	0.116	0.078
HGFA	4.71 ± 1.39	4.43 ± 2.33	0.354	0.422
LBP	37.2 ± 14.6	30.9 ± 14.4	0.209	0.200
Prothrombin	1463 ± 323	1480 ± 327	0.870	0.967
SeP	162 ± 56.9	144 ± 39.6	0.660	0.543
TBG	117 ± 15.5	119 ± 32.3	0.880	0.898
Transthyretin	7718 ± 1870	6338 ± 1801	0.026	0.038

*MDD* major depressive disorder, *SD* standard deviation

^a^Multivariable P-value from partial Spearman correlation analysis including age and gender

### Plasma protein concentrations in MDD patients treated with SSRIs

In patients treated with SSRIs, the concentrations of HGFA and LBP were numerically different between responders and nonresponders at baseline (5.44 vs. 4.00 fmol/μL, uni-P = 0.141, multi-P = 0.193 for HGFA; 28.8 vs. 33.6 fmol/μL, uni-P = 0.362, multi-P = 0.053 for LBP; Table 4 and Fig. 2b). TBG levels were increased in responders after SSRI treatment (118 vs. 129 fmol/μL, uni-P = 0.090). However, these differences according to responsiveness to SSRIs and changes according to SSRIs treatment of plasma protein did not reach statistical significance.

**Table 4:** Plasma protein concentrations (fmol/μL) at baseline and at 6 weeks after SSRIs treatment

Protein	Responder			Nonresponder			Responsiveness	
	Mean ± SD		Uni-P^a^	Mean ± SD		Uni-P^a^	Uni-P^b^	Multi-P^c^
	At baseline	At 6 week		At baseline	At 6 week			
N	19			16				
AGP1	8325 ± 2672	7916 ± 2084	0.455	8014 ± 3334	7888 ± 2856	0.433	0.540	0.954
HGFA	5.44 ± 3.60	4.50 ± 1.64	0.374	4.00 ± 1.55	4.01 ± 1.53	0.968	0.141	0.193
LBP	28.8 ± 13.2	32.0 ± 9.58	0.290	33.6 ± 17.6	30.0 ± 9.54	0.706	0.362	0.053
Prothrombin	1485 ± 294	1499 ± 172	0.728	1450 ± 316	1405 ± 325	0.744	0.733	0.294
SeP	137 ± 37.1	148 ± 16.5	0.237	129 ± 36.9	122 ± 38.2	0.464	0.417	0.465
TBG	118 ± 27.9	129 ± 24.9	0.090	128 ± 36.9	124 ± 24.9	0.336	0.354	0.370
Transthyretin	6105 ± 1689	6448 ± 1444	0.361	6016 ± 1681	5864 ± 1867	0.585	0.876	0.351

*HAM-D* Hamilton Depression Rating Scale, *SD* standard deviation, *SSRIs* selective serotonin reuptake inhibitors

^a^P-value from Wilcoxon signed rank test or paired T-test between at baseline and at 6 weeks in each response group

^b^P-value from Mann–Whitney test or T-test between responders and nonresponders at baseline

^c^Multivariable P-value from partial Spearman correlation analysis including clinical variables with P < 0.020 (HAM-D score at baseline, number of episode, and duration of current episode)

### Plasma protein concentrations in MDD patients treated with mirtazapine

In patients treated with mirtazapine, although plasma protein concentration was higher in responders than that in nonresponders for HGFA (4.48 vs. 3.79 fmol/μL, uni-P = 0.191) and lower in responders for prothrombin (1450 vs. 1528 fmol/μL, uni-P = 0.057) at baseline, these differences did not show statistical significance (Table 5 and Fig. 2c).

**Table 5:** Plasma protein concentrations (fmol/μL) at baseline and at 6 weeks after mirtazapine treatment

Protein	Responder			Nonresponder			Responsiveness	
	Mean ± SD		Uni-P^a^	Mean ± SD		Uni-P^a^	Uni-P^b^	Multi-P^c^
	At baseline	At 6 week		At baseline	At 6 week			
N	22			21				
AGP1	7872 ± 2719	6967 ± 1610	0.087	7360 ± 3620	6460 ± 1356	0.448	0.280	0.462
HGFA	4.48 ± 1.84	4.21 ± 1.36	0.449	3.79 ± 1.51	4.73 ± 1.83	0.015	0.191	0.270
LBP	31.4 ± 16.2	33.4 ± 12.7	0.219	30.0 ± 10.7	30.8 ± 9.64	0.629	0.585	0.772
Prothrombin	1450 ± 284	1549 ± 383	0.302	1528 ± 412	1486 ± 395	0.657	0.057	0.246
SeP	146 ± 26.2	154 ± 40.4	0.389	157 ± 51.4	158 ± 40.1	0.974	0.397	0.395
TBG	120 ± 28.6	139 ± 38.3	0.007	112 ± 36.2	118 ± 22.5	0.725	0.552	0.511
Transthyretin	6294 ± 1783	6968 ± 2063	0.095	6841 ± 2016	7425 ± 1954	0.209	0.351	0.379

*HAM-D* Hamilton Depression Rating Scale, *SD* standard deviation

^a^P-value from Wilcoxon signed rank test or paired T-test between at baseline and at 6 weeks in each response group

^b^P-value from Mann–Whitney test or T-test between responders and nonresponders at baseline

^c^Multivariable P-value from partial Spearman correlation analysis including clinical variables with P < 0.020 (HAM-D score at baseline, and duration of current episode)

In analysis for changes according to mirtazapine treatment, TBG was increased after mirtazapine treatment in responders (120 vs. 139 fmol/μL, uni-P = 0.007). On the other hand, TBG did not show statistically significant changes according to mirtazapine treatment in nonresponders.

## Discussion

In this study, we performed MRM-based proteomic analysis using peripheral blood specimens at baseline and at 6 weeks after antidepressant treatment in MDD patients and from healthy individuals. We also quantified proteins using stable isotope labeled peptide. We identified a difference in plasma transthyretin concentration between MDD patients and healthy individuals, and a change in plasma TBG concentration related to mirtazapine treatment.

The diagnosis of MDD is primarily based on clinical features, not objective biomarkers. Biomarkers of diagnosis are potential candidates for disease monitoring. Our study showed lower transthyretin level in MDD patients than that in healthy individuals. Transthyretin is a carrier protein which transports thyroxin (T4) and retinol in plasma and CSF [31]. It has been reported that transthyretin is associated with neurodegenerative disorders and psychiatric condition including major depressive disorder [31]. Transthyretin level has been found to be low in CSF from patients with Alzheimer’s Disease [31–33]. Correlation between plasma transthyretin level and severity and progression of Alzheimer’s disease has also been reported [34]. Previous study has also reported that CSF transthyretin level is lower in depressive patients compared to that in healthy controls [35, 36]. Serum transthyretin level is also lower in patients with post-stroke depression than that in patients without post-stroke depression [37]. In preclinical studies using animal model of depression, administration of sodium butyrate, a histone deacetylase inhibitor with antidepressant-like effect, has resulted upregulation of transthyretin RNA level [38, 39]. Low plasma transthyretin level in MDD patients in our study could be meaningful in the aspect of that this is the first demonstration using plasma from MDD patients. Although conflicting findings have been reported in preclinical studies [40] and whether transthyretin alteration is a cause or consequence of MDD remains unclear, it has been suggested that low transthyretin level can lower thyroid hormone availability which may lead to depression [35, 39].

In analysis for identifying proteins whose levels are changed after antidepressant treatment, TBG level was found to be elevated after mirtazapine treatment only in responders. TBG has not been measured in MDD yet. Its changes in response to antidepressant treatment has not been investigated in previous studies either. Only one study has evaluated TBG as a predictor of perinatal syndromal depression based on relationship between hypothalamic-pituitary-thyroid axis abnormality and pregnancy-related depression [41]. TBG is one of the major thyroid-hormone binding proteins, along with transthyretin and albumin. In the present study, TBG level was increased in responders after treatment with SSRIs (uni-P = 0.090) and transthyretin level was increased in responders after treatment with mirtazapine (uni-P = 0.095). Although these increases did not show statistical significance, they were in line with the difference of transthyretin levels between MDD patients and controls and changes of TBG levels in responders to mirtazapine. These findings suggest that alterations and changes of transportation of thyroid hormones can mediate the effect of antidepressants and clinical manifestation of MDD. These findings have not been reported in previous studies. They need to be confirmed by further studies.

When compared between responders and nonresponders to both SSRIs and mirtazapine, HGFA showed higher concentrations in responders. HGFA is involved in the HGF-Met pathway correlated in tissue protection, regeneration, and anti-fibrosis/inflammation [42]. With regard to MDD, it was suggested that the HGFA concentration correlated with severity and symptoms of depression in previous studies [43, 44]. HGFA as a marker that predicts treatment response should be demonstrated further analysis because the correlation between HGFA concentration and treatment responsiveness was not statistically significant in our study.

We performed proteomic analysis using pre- and post-plasma specimens after SSRIs and mirtazapine treatment from MDD patients and healthy individuals. Our results showed alterations and changes of plasma proteins including transthyretin and TBG. These findings are meaningful because there are few studies about changes of protein expression after antidepressant treatment or differences at baseline associated with responsiveness. Our findings have some limitations. First, performed evaluation of our method to detect plasma proteins through several experimental stages. However, we did not validate our results using independent study population. Therefore, findings of this study should be considered as exploratory findings. Further validation study is required.

## Conclusions

We identified proteins including transthyretin and TBG, which were altered in MDD after antidepressant treatment using proteomic analysis of plasma specimens. Our findings suggest that alteration and change in transportation of thyroid hormones can be related to the effect of antidepressants and clinical manifestation of MDD.

## Supplementary Information

**Additional file 1: Table S1.** 111 candidate proteins for proteomic analysis.

## Data Availability

All data in our study are available upon request.

## Abbreviations

MDD: Major depressive disorder
MRM: Multiple reaction monitoring
SSRIs: Selective serotonin reuptake inhibitors
TBG: Thyroxine-binding globulin
CSF: Cerebrospinal fluid
ECT: Electroconvulsive therapy
HAM-D: Hamilton rating scale for depression
AGP1: Alpha-1-acid glycoprotein 1
HGFA: Hepatocyte growth factor activator
LBP: Lipopolysaccharide-binding protein
SeP: Prothrombin, selenoprotein P
TBG: Thyroxine-binding globulin
S/N: Signal-to-noise ratio
T4: Thyroxin
