# Supplementary material for: Alteration of transthyretin and thyroxine-binding globulin in major depressive disorder: multiple reaction monitoring-based proteomic analysis
Source: J Transl Med. 2021 Jan 15;19:34. doi: 10.1186/s12967-021-02702-y (PMC7811235; doi:10.1186/s12967-021-02702-y)
Supplement: Supplementary file 1 — Additional file 1: Table S1. 111 candidate proteins for proteomic analysis. [file 12967_2021_2702_MOESM1_ESM.docx]

**Additional file 1: Table S1.** 111 candidate proteins for proteomic analysis

| No. | Gene symbol | Accession no. | Protein name | Reference |
| --- | --- | --- | --- | --- |
| 1 | A2M | P01023 | Alpha-2-macroglobulin | [[1](#_ENREF_1)] |
| 2 | ADRA1A | P35348 | Alpha-1A adrenergic receptor | [[2](#_ENREF_2)] |
| 3 | ADRB1 | P08588 | Beta-1 adrenergic receptor | [[2](#_ENREF_2)] |
| 4 | APOA1 | P02647 | Apolipoprotein A-I | [[1](#_ENREF_1), [3](#_ENREF_3)] |
| 5 | B2M | P61769 | Beta-2-microglobulin | [[1](#_ENREF_1)] |
| 6 | BDNF | P23560 | Brain-derived neurotrophic factor | [[2-5](#_ENREF_2)] |
| 7 | CCL11 | P51671 | Eotaxin | [[6](#_ENREF_6)] |
| 8 | CCL2 | P13500 | C-C motif chemokine 2 | [[2](#_ENREF_2), [6](#_ENREF_6)] |
| 9 | CD40LG | P29965 | CD40 ligand | [[7](#_ENREF_7)] |
| 10 | CFD | P00746 | Complement factor D | [[8](#_ENREF_8)] |
| 11 | CNR1 | P21554 | Cannabinoid receptor 1 | [[2](#_ENREF_2)] |
| 12 | COMT | P21964 | Catechol O-methyltransferase | [[2](#_ENREF_2)] |
| 13 | COPA | P53621 | Coatomer subunit alpha | [[9](#_ENREF_9)] |
| 14 | CPLX2 | Q6PUV4 | Complexin-2 | [[2](#_ENREF_2)] |
| 15 | CRP | P02741 | C-reactive protein | [[1](#_ENREF_1), [3](#_ENREF_3)] |
| 16 | CSF2 | P04141 | Granulocyte-macrophage colony-stimulating factor | [[6](#_ENREF_6)] |
| 17 | CTSB | P07858 | Cathepsin B | [[9](#_ENREF_9)] |
| 18 | CXCL8 | P10145 | Interleukin-8 | [[6](#_ENREF_6)] |
| 19 | DBH | P09172 | Dopamine beta-hydroxylase | [[2](#_ENREF_2)] |
| 20 | DDC | P20711 | Aromatic-L-amino-acid decarboxylase | [[10](#_ENREF_10)] |
| 21 | DRD1 | P21728 | D(1A) dopamine receptor | [[2](#_ENREF_2)] |
| 22 | DRD2 | P14416 | D(2) dopamine receptor | [[2](#_ENREF_2)] |
| 23 | DRD3 | P35462 | D(3) dopamine receptor | [[2](#_ENREF_2)] |
| 24 | DRD4 | P21917 | D(4) dopamine receptor | [[2](#_ENREF_2)] |
| 25 | DTNBP1 | Q96EV8 | Dysbindin | [[2](#_ENREF_2)] |
| 26 | F2 | P00734 | Prothrombin | [[7](#_ENREF_7)] |
| 27 | F7 | P08709 | Coagulation factor VII | [[7](#_ENREF_7)] |
| 28 | FBLN1 | P23142 | Fibulin-1 | [[11](#_ENREF_11), [12](#_ENREF_12)] |
| 29 | FETUB | Q9UGM5 | Fetuin-B | [[13](#_ENREF_13)] |
| 30 | FGF2 | P09038 | Fibroblast growth factor 2 | [[14](#_ENREF_14), [15](#_ENREF_15)] |
| 31 | FGFR1 | P11362 | Fibroblast growth factor receptor 1 | [[15](#_ENREF_15), [16](#_ENREF_16)] |
| 32 | FGFR2 | P21802 | Fibroblast growth factor receptor 2 | [[14](#_ENREF_14), [17](#_ENREF_17)] |
| 33 | GABRA1 | P14867 | Gamma-aminobutyric acid receptor subunit alpha-1 | [[2](#_ENREF_2), [18](#_ENREF_18)] |
| 34 | GABRA2 | P47869 | Gamma-aminobutyric acid receptor subunit alpha-2 | [[2](#_ENREF_2)] |
| 35 | GABRA3 | P34903 | Gamma-aminobutyric acid receptor subunit alpha-3 | [[2](#_ENREF_2)] |
| 36 | GABRA5 | P31644 | Gamma-aminobutyric acid receptor subunit alpha-5 | [[2](#_ENREF_2)] |
| 37 | GABRA6 | Q16445 | Gamma-aminobutyric acid receptor subunit alpha-6 | [[2](#_ENREF_2)] |
| 38 | GAD1 | Q99259 | Glutamate decarboxylase 1 | [[2](#_ENREF_2), [19](#_ENREF_19)] |
| 39 | GAD2 | Q05329 | Glutamate decarboxylase 2 | [[2](#_ENREF_2)] |
| 40 | GDNF | P39905 | Glial cell line-derived neurotrophic factor | [[2](#_ENREF_2)] |
| 41 | GH1 | P01241 | Somatotropin | [[1](#_ENREF_1)] |
| 42 | GLDC | P23378 | Glycine dehydrogenase (decarboxylating), mitochondrial | [[20](#_ENREF_20)] |
| 43 | GLUL | P15104 | Glutamine synthetase | [[21](#_ENREF_21)] |
| 44 | GNAL | P38405 | Guanine nucleotide-binding protein G(olf) subunit alpha | [[2](#_ENREF_2)] |
| 45 | GNAS | Q5JWF2 | Guanine nucleotide-binding protein G(s) subunit alpha isoforms short | [[2](#_ENREF_2)] |
| 46 | GP1BA | P07359 | Platelet glycoprotein Ib alpha chain | [[22](#_ENREF_22)] |
| 47 | GRIA1 | P42261 | Glutamate receptor 1 | [[2](#_ENREF_2), [21](#_ENREF_21)] |
| 48 | GRIK3 | Q13003 | Glutamate receptor ionotropic, kainate 3 | [[2](#_ENREF_2)] |
| 49 | GRIN1 | Q05586 | Glutamate receptor ionotropic, NMDA 1 | [[2](#_ENREF_2)] |
| 50 | GRIN2A | Q12879 | Glutamate receptor ionotropic, NMDA 2A | [[2](#_ENREF_2)] |
| 51 | GRIN2B | Q13224 | Glutamate receptor ionotropic, NMDA 2B | [[2](#_ENREF_2)] |
| 52 | GRM1 | Q13255 | Metabotropic glutamate receptor 1 | [[4](#_ENREF_4), [23](#_ENREF_23)] |
| 53 | GSK3B | P49841 | Glycogen synthase kinase-3 beta | [[2](#_ENREF_2), [24](#_ENREF_24), [25](#_ENREF_25)] |
| 54 | HBA1 | P69905 | Hemoglobin subunit alpha | [[26](#_ENREF_26)] |
| 55 | HCRT | O43612 | Orexin | [[27](#_ENREF_27)] |
| 56 | HGFAC | Q04756 | Hepatocyte growth factor activator | [[28](#_ENREF_28)] |
| 57 | HOMER2 | Q9NSB8 | Homer protein homolog 2 | [[4](#_ENREF_4)] |
| 58 | HTR1A | P08908 | 5-hydroxytryptamine receptor 1A | [[2](#_ENREF_2)] |
| 59 | HTR1B | P28222 | 5-hydroxytryptamine receptor 1B | [[2](#_ENREF_2)] |
| 60 | HTR2A | P28223 | 5-hydroxytryptamine receptor 2A | [[2](#_ENREF_2)] |
| 61 | HTR2C | P28335 | 5-hydroxytryptamine receptor 2C | [[2](#_ENREF_2)] |
| 62 | HTR3A | P46098 | 5-hydroxytryptamine receptor 3A | [[2](#_ENREF_2)] |
| 63 | HTR5A | P47898 | 5-hydroxytryptamine receptor 5A | [[2](#_ENREF_2)] |
| 64 | HTR6 | P50406 | 5-hydroxytryptamine receptor 6 | [[2](#_ENREF_2)] |
| 65 | HTT | P42858 | Huntingtin | [[2](#_ENREF_2)] |
| 66 | IFNG | P01579 | Interferon gamma | [[6](#_ENREF_6), [29](#_ENREF_29)] |
| 67 | IGFBP3 | P17936 | Insulin-like growth factor-binding protein 3 | [[30](#_ENREF_30)] |
| 68 | IL12A | O60595 | Interleukin-12 subunit alpha | [[6](#_ENREF_6)] |
| 69 | IL15 | P40933 | Interleukin-15 | [[6](#_ENREF_6)] |
| 70 | IL1A | P01583 | Interleukin-1 alpha | [[3](#_ENREF_3), [6](#_ENREF_6)] |
| 71 | IL1B | P01584 | Interleukin-1 beta | [[2](#_ENREF_2), [6](#_ENREF_6), [7](#_ENREF_7)] |
| 72 | IL2 | P60568 | Interleukin-2 | [[6](#_ENREF_6)] |
| 73 | IL4 | P05112 | Interleukin-4 | [[6](#_ENREF_6)] |
| 74 | IL6 | P05231 | Interleukin-6 | [[2](#_ENREF_2), [6](#_ENREF_6), [7](#_ENREF_7)] |
| 75 | IL7 | P13232 | Interleukin-7 | [[6](#_ENREF_6)] |
| 76 | INS | P01308 | Insulin | [[1](#_ENREF_1)] |
| 77 | LBP | P18428 | Lipopolysaccharide-binding protein | [[31](#_ENREF_31)] |
| 78 | MAOA | P21397 | Amine oxidase [flavin-containing] A | [[2](#_ENREF_2)] |
| 79 | MAOB | P27338 | Amine oxidase [flavin-containing] B | [[2](#_ENREF_2)] |
| 80 | MMP2 | P08253 | 72 kDa type IV collagenase | [[1](#_ENREF_1)] |
| 81 | MMP9 | P14780 | Matrix metalloproteinase-9 | [[1](#_ENREF_1)] |
| 82 | NOS1 | P29475 | Nitric oxide synthase, brain | [[2](#_ENREF_2)] |
| 83 | NTRK2 | Q16620 | BDNF/NT-3 growth factors receptor | [[2](#_ENREF_2), [17](#_ENREF_17)] |
| 84 | OPRM1 | P35372 | Mu-type opioid receptor | [[2](#_ENREF_2)] |
| 85 | ORM1 | P02763 | Alpha-1-acid glycoprotein 1 | [[32](#_ENREF_32), [33](#_ENREF_33)] |
| 86 | P2RX7 | Q99572 | P2X purinoceptor 7 | [[2](#_ENREF_2), [17](#_ENREF_17)] |
| 87 | PRKACA | P17612 | cAMP-dependent protein kinase catalytic subunit alpha | [[2](#_ENREF_2)] |
| 88 | PROC | P04070 | Vitamin K-dependent protein C | [[34](#_ENREF_34)] |
| 89 | PROS1 | P07225 | Vitamin K-dependent protein S | [[34](#_ENREF_34)] |
| 90 | PROZ | P22891 | Vitamin K-dependent protein Z | [[34](#_ENREF_34)] |
| 91 | PRPF19 | Q9UMS4 | Pre-mRNA-processing factor 19 | [[4](#_ENREF_4), [16](#_ENREF_16), [17](#_ENREF_17)] |
| 92 | SELENOP | P49908 | Selenoprotein P | [[35](#_ENREF_35)] |
| 93 | SELP | P16109 | P-selectin | [[7](#_ENREF_7)] |
| 94 | SERPINA7 | P05543 | Thyroxine-binding globulin | [[36](#_ENREF_36)] |
| 95 | SERPINE1 | P05121 | Plasminogen activator inhibitor 1 | [[1](#_ENREF_1), [2](#_ENREF_2)] |
| 96 | SHBG | P04278 | Sex hormone-binding globulin | [[37](#_ENREF_37)] |
| 97 | SLC18A2 | Q05940 | Synaptic vesicular amine transporter | [[2](#_ENREF_2)] |
| 98 | SLC1A2 | P43004 | Excitatory amino acid transporter 2 | [[21](#_ENREF_21)] |
| 99 | SLC1A3 | P43003 | Excitatory amino acid transporter 1 | [[21](#_ENREF_21)] |
| 100 | SLC1A4 | P43007 | Neutral amino acid transporter A | [[2](#_ENREF_2)] |
| 101 | SLC6A1 | P30531 | Sodium- and chloride-dependent GABA transporter 1 | [[2](#_ENREF_2)] |
| 102 | SLC6A3 | Q01959 | Sodium-dependent dopamine transporter | [[2](#_ENREF_2), [38](#_ENREF_38)] |
| 103 | TACR1 | P25103 | Substance-P receptor | [[2](#_ENREF_2)] |
| 104 | TF | P02787 | Serotransferrin | [[3](#_ENREF_3), [17](#_ENREF_17)] |
| 105 | TH | P07101 | Tyrosine 3-monooxygenase | [[2](#_ENREF_2)] |
| 106 | TNF | P01375 | Tumor necrosis factor | [[2](#_ENREF_2), [7](#_ENREF_7)] |
| 107 | TNFRSF1B | P20333 | Tumor necrosis factor receptor superfamily member 1B | [[1](#_ENREF_1)] |
| 108 | TNPO1 | Q92973 | Transportin-1 | [[6](#_ENREF_6)] |
| 109 | TPH1 | P17752 | Tryptophan 5-hydroxylase 1 | [[2](#_ENREF_2)] |
| 110 | TTR | P02766 | Transthyretin | [[39](#_ENREF_39)] |
| 111 | ZKSCAN1 | P17029 | Zinc finger protein with KRAB and SCAN domains 1 | [[16](#_ENREF_16), [17](#_ENREF_17)] |

**References**

1. Domenici E, Wille DR, Tozzi F, Prokopenko I, Miller S, McKeown A, Brittain C, Rujescu D, Giegling I, Turck CW, Holsboer F, Bullmore ET, Middleton L, Merlo-Pich E, Alexander RC, Muglia P. Plasma protein biomarkers for depression and schizophrenia by multi analyte profiling of case-control collections. PLoS One. 2010;5:e9166.

2. Kao CF, Fang YS, Zhao Z, Kuo PH. Prioritization and evaluation of depression candidate genes by combining multidimensional data resources. PLoS One. 2011;6:e18696.

3. Carboni L, Becchi S, Piubelli C, Mallei A, Giambelli R, Razzoli M, Mathe AA, Popoli M, Domenici E. Early-life stress and antidepressants modulate peripheral biomarkers in a gene-environment rat model of depression. Prog Neuropsychopharmacol Biol Psychiatry. 2010;34:1037-48.

4. Altar CA, Vawter MP, Ginsberg SD. Target identification for CNS diseases by transcriptional profiling. Neuropsychopharmacology. 2009;34:18-54.

5. Juhasz G, Dunham JS, McKie S, Thomas E, Downey D, Chase D, Lloyd-Williams K, Toth ZG, Platt H, Mekli K, Payton A, Elliott R, Williams SR, Anderson IM, Deakin JF. The CREB1-BDNF-NTRK2 pathway in depression: multiple gene-cognition-environment interactions. Biol Psychiatry. 2011;69:762-71.

6. Simon NM, McNamara K, Chow CW, Maser RS, Papakostas GI, Pollack MH, Nierenberg AA, Fava M, Wong KK. A detailed examination of cytokine abnormalities in Major Depressive Disorder. Eur Neuropsychopharmacol. 2008;18:230-3.

7. Leo R, Di Lorenzo G, Tesauro M, Razzini C, Forleo GB, Chiricolo G, Cola C, Zanasi M, Troisi A, Siracusano A, Lauro R, Romeo F. Association between enhanced soluble CD40 ligand and proinflammatory and prothrombotic states in major depressive disorder: pilot observations on the effects of selective serotonin reuptake inhibitor therapy. J Clin Psychiatry. 2006;67:1760-6.

8. Savitz J, Frank MB, Victor T, Bebak M, Marino JH, Bellgowan PS, McKinney BA, Bodurka J, Kent Teague T, Drevets WC. Inflammation and neurological disease-related genes are differentially expressed in depressed patients with mood disorders and correlate with morphometric and functional imaging abnormalities. Brain Behav Immun. 2013;31:161-71.

9. Sequeira A, Gwadry FG, Ffrench-Mullen JM, Canetti L, Gingras Y, Casero RA, Jr., Rouleau G, Benkelfat C, Turecki G. Implication of SSAT by gene expression and genetic variation in suicide and major depression. Arch Gen Psychiatry. 2006;63:35-48.

10. Matthes S, Mosienko V, Bashammakh S, Alenina N, Bader M. Tryptophan hydroxylase as novel target for the treatment of depressive disorders. Pharmacology. 2010;85:95-109.

11. Gottschalk MG, Cooper JD, Chan MK, Bot M, Penninx BW, Bahn S. Serum biomarkers predictive of depressive episodes in panic disorder. J Psychiatr Res. 2016;73:53-62.

12. Shen L, Liao L, Chen C, Guo Y, Song D, Wang Y, Chen Y, Zhang K, Ying M, Li S, Liu Q, Ni J. Proteomics Analysis of Blood Serums from Alzheimer's Disease Patients Using iTRAQ Labeling Technology. J Alzheimers Dis. 2017;56:361-78.

13. Ramsey JM, Cooper JD, Bot M, Guest PC, Lamers F, Weickert CS, Penninx BW, Bahn S. Sex Differences in Serum Markers of Major Depressive Disorder in the Netherlands Study of Depression and Anxiety (NESDA). PLoS One. 2016;11:e0156624.

14. Evans SJ, Choudary PV, Neal CR, Li JZ, Vawter MP, Tomita H, Lopez JF, Thompson RC, Meng F, Stead JD, Walsh DM, Myers RM, Bunney WE, Watson SJ, Jones EG, Akil H. Dysregulation of the fibroblast growth factor system in major depression. Proc Natl Acad Sci U S A. 2004;101:15506-11.

15. Gaughran F, Payne J, Sedgwick PM, Cotter D, Berry M. Hippocampal FGF-2 and FGFR1 mRNA expression in major depression, schizophrenia and bipolar disorder. Brain Res Bull. 2006;70:221-7.

16. Tochigi M, Iwamoto K, Bundo M, Sasaki T, Kato N, Kato T. Gene expression profiling of major depression and suicide in the prefrontal cortex of postmortem brains. Neurosci Res. 2008;60:184-91.

17. Aston C, Jiang L, Sokolov BP. Transcriptional profiling reveals evidence for signaling and oligodendroglial abnormalities in the temporal cortex from patients with major depressive disorder. Mol Psychiatry. 2005;10:309-22.

18. Sequeira A, Klempan T, Canetti L, ffrench-Mullen J, Benkelfat C, Rouleau GA, Turecki G. Patterns of gene expression in the limbic system of suicides with and without major depression. Mol Psychiatry. 2007;12:640-55.

19. Hettema JM, An SS, Neale MC, Bukszar J, van den Oord EJ, Kendler KS, Chen X. Association between glutamic acid decarboxylase genes and anxiety disorders, major depression, and neuroticism. Mol Psychiatry. 2006;11:752-62.

20. Ji Y, Hebbring S, Zhu H, Jenkins GD, Biernacka J, Snyder K, Drews M, Fiehn O, Zeng Z, Schaid D, Mrazek DA, Kaddurah-Daouk R, Weinshilboum RM. Glycine and a glycine dehydrogenase (GLDC) SNP as citalopram/escitalopram response biomarkers in depression: pharmacometabolomics-informed pharmacogenomics. Clin Pharmacol Ther. 2011;89:97-104.

21. Choudary PV, Molnar M, Evans SJ, Tomita H, Li JZ, Vawter MP, Myers RM, Bunney WE, Jr., Akil H, Watson SJ, Jones EG. Altered cortical glutamatergic and GABAergic signal transmission with glial involvement in depression. Proc Natl Acad Sci U S A. 2005;102:15653-8.

22. Walsh MT, Dinan TG, Condren RM, Ryan M, Kenny D. Depression is associated with an increase in the expression of the platelet adhesion receptor glycoprotein Ib. Life Sci. 2002;70:3155-65.

23. Menke A, Samann P, Kloiber S, Czamara D, Lucae S, Hennings J, Heck A, Kohli MA, Czisch M, Muller-Myhsok B, Holsboer F, Binder EB. Polymorphisms within the metabotropic glutamate receptor 1 gene are associated with depression phenotypes. Psychoneuroendocrinology. 2012;37:565-75.

24. Diniz BS, Talib LL, Joaquim HP, de Paula VR, Gattaz WF, Forlenza OV. Platelet GSK3B activity in patients with late-life depression: marker of depressive episode severity and cognitive impairment? World J Biol Psychiatry. 2011;12:216-22.

25. Oh DH, Park YC, Kim SH. Increased glycogen synthase kinase-3beta mRNA level in the hippocampus of patients with major depression: a study using the stanley neuropathology consortium integrative database. Psychiatry Investig. 2010;7:202-7.

26. Bachle C, Lange K, Stahl-Pehe A, Castillo K, Holl RW, Giani G, Rosenbauer J. Associations between HbA1c and depressive symptoms in young adults with early-onset type 1 diabetes. Psychoneuroendocrinology. 2015;55:48-58.

27. Rotter A, Asemann R, Decker A, Kornhuber J, Biermann T. Orexin expression and promoter-methylation in peripheral blood of patients suffering from major depressive disorder. J Affect Disord. 2011;131:186-92.

28. Chan MK, Cooper JD, Bot M, Birkenhager TK, Bergink V, Drexhage HA, Steiner J, Rothermundt M, Penninx BW, Bahn S. Blood-based immune-endocrine biomarkers of treatment response in depression. J Psychiatr Res. 2016;83:249-59.

29. Oxenkrug G, Perianayagam M, Mikolich D, Requintina P, Shick L, Ruthazer R, Zucker D, Summergrad P. Interferon-gamma (+874) T/A genotypes and risk of IFN-alpha-induced depression. J Neural Transm (Vienna). 2011;118:271-4.

30. Wang Y, Huang M, Jiao JT, Wu YL, Ouyang TH, Huang J, Liu SS, Li CL. Relationship between concentrations of IGF-1 and IGFBP-3 and preoperative depression risk, and effect of psychological intervention on outcomes of high-grade glioma patients with preoperative depression in a 2-year prospective study. Med Oncol. 2014;31:921.

31. Schahab S, Heun R, Schmitz S, Maier W, Kolsch H. Association of polymorphism in the transcription factor LBP-1c/CP2/LSF gene with Alzheimer's disease and major depression. Dement Geriatr Cogn Disord. 2006;22:95-8.

32. Adeoye OM, Ferrell RE, Kirshner MA, Mulsant BH, Seligman K, Begley AE, Reynolds CF, 3rd, Pollock BG. alpha1-acid glycoprotein in late-life depression: relationship to medical burden and genetics. J Geriatr Psychiatry Neurol. 2003;16:235-9.

33. Harley J, Roberts R, Joyce P, Mulder R, Luty S, Frampton C, Kennedy M. Orosomucoid influences the response to antidepressants in major depressive disorder. J Psychopharmacol. 2010;24:531-5.

34. Rubio-Lopez N, Morales-Suarez-Varela M, Pico Y, Livianos-Aldana L, Llopis-Gonzalez A. Nutrient Intake and Depression Symptoms in Spanish Children: The ANIVA Study. Int J Environ Res Public Health. 2016;13.

35. Pasco JA, Jacka FN, Williams LJ, Evans-Cleverdon M, Brennan SL, Kotowicz MA, Nicholson GC, Ball MJ, Berk M. Dietary selenium and major depression: a nested case-control study. Complement Ther Med. 2012;20:119-23.

36. Pedersen C, Leserman J, Garcia N, Stansbury M, Meltzer-Brody S, Johnson J. Late pregnancy thyroid-binding globulin predicts perinatal depression. Psychoneuroendocrinology. 2016;65:84-93.

37. Wdowiak A, Bien A, Iwanowicz-Palus G, Makara-Studzinska M, Bojar I. Impact of emotional disorders on semen quality in men treated for infertility. Neuro Endocrinol Lett. 2017;38:50-8.

38. Pinsonneault JK, Han DD, Burdick KE, Kataki M, Bertolino A, Malhotra AK, Gu HH, Sadee W. Dopamine transporter gene variant affecting expression in human brain is associated with bipolar disorder. Neuropsychopharmacology. 2011;36:1644-55.

39. Fleming CE, Nunes AF, Sousa MM. Transthyretin: more than meets the eye. Prog Neurobiol. 2009;89:266-76.
